# Repetitive transcranial magnetic stimulation (rTMS) improves behavioral and biochemical deficits in levodopa-induced dyskinetic rats model

**DOI:** 10.18632/oncotarget.11587

**Published:** 2016-08-24

**Authors:** Maowen Ba, Min Kong, Lina Guan, Maoli Yi, Hongli Zhang

**Affiliations:** ^1^ Department of Neurology, Yantai Yuhuangding Hospital Affiliated to Qingdao Medical University, Shandong, PR China; ^2^ Department of Neurology, Yantaishan Hospital, Yantai City, Shandong, PR China; ^3^ Department of Laboratory, Yuhuangding Hospital Affiliated to Qingdao Medical University, Shandong, PR China; ^4^ Department of Endocrinology, Ruijin Hospital Affiliated To Shanghai Jiaotong University School of Medicine, Shanghai, China

**Keywords:** dyskinesia, repetitive transcranial magnetic stimulation (rTMS), dopamine, NR2B, phosphorylation, Gerotarget

## Abstract

Fluctuations of dopamine levels and upregulations of NR2B tyrosine phosphorylation in the striatum have been connected with levodopa (L-dopa)-induced dyskinesia (LID) in Parkinson's disease (PD). Repetitive transcranial magnetic stimulation (rTMS) is one of the noninvasive and potential method treating dyskinesia. Yet, the effect of rTMS on the above key pathological events remains unclear. In this study, we gave L-dopa treatment intraperitoneally for 22 days to 6-hydroxydopamine-lesioned PD rats to prepare LID rats model, and subsequently applied rTMS daily for 3 weeks to LID rats model. The effect of rTMS on abnormal involuntary movements (AIMs) was assessed. After ending the experiments, we further determined tyrosine hydroxylase (TH)-positive dopaminergic neurons number by immunohistochemistry, dopamine levels by HPLC, glial cell line-derived neurotrophic factor (GDNF) levels by ELISA, NR2B tyrosine phosphorylation and interactions of NR2B with Fyn by immunoblotting and immunoprecipitation. The results demonstrated that rTMS obviously attenuated AIMs scores, reduced the loss of nigral dopaminergic neurons and the fluctuations of striatal dopamine levels. Meanwhile, rTMS significantly increased the expression of GDNFwhich couldrestore the damage of dopaminergic neurons. Additionally, rTMS also reduced the levels of the NR2B tyrosine phosphorylation andits interactions with Fyn in the lesioned striatum of LID rats model. Thus, these data indicate that rTMS can provide benefit for the therapy of LID by improving the key biochemical deficits related to dyskinesia.

## INTRODUCTION

Dyskinesia is one of the most common motor complications in Parkinson's disease (PD) in the long-term levodopa (L-dopa) therapy process [1, 2]. Striatal dopamine decrease and receptor denervation by progressive loss of dopaminergic neurons as well as L-dopa therapy are considered vital contributors [3-7]. The fluctuations of dopamine (DA) levels lead to the striatal dopamine receptors aberant stimulation, which can further activate iontotropic glutamatergic receptors [4, 8, 9]. In this regard, abnormal function of striatal glutamatergic *N*-methyl-*D*-aspartate receptor (NMDAR) has been implicated in the pathogenesis of PD and L-dopa-induced dyskinesia (LID) [10-12]. Recent evidences including our research reports indicated that chronic dopaminergic treatment lead to the overactivation of striatal NMDAR by enhanced NR2B tyrosine phosphorylation dependent on the interactions of NR2B with Fyn tyrosine kinase, which were proved to correlate with LID [13-17]. Thus, these abnormal pathological events may also become effective targets for treating dyskinesia.

Repetitive transcranial magnetic stimulation (rTMS) is one of the broadly-used, noninvasive and potential method in the treatment of many neurological diseases [18]. Clinical and preclinical research indicates that rTMS is effective in improving PD and dyskinesia. It appears that rTMS can mediate the neuroplasticity and decrease an imbalance between excitatory and inhibitory inputs from the basal ganglia to motor and premotor areas in dyskinetic PD patients [19-23]. In spite of great research effort, the underlying precise molecular mechanism by which rTMS acts as one antidyskinetic treatment method is still less well understood. Based on the LID rats model, the present study was carried out to further clarify the neurorestorative efficiency and molecular mechanisms of rTMS treatment in LID. After a low frequency rTMS treatment in dyskinetic rats model, changes were investigated in the expression of dyskinetic behavior, nigral dopaminergic neurons and striatal DA levels, NR2B tyrosine phosphorylation and the interactions of NR2B with Fyn, respectively.

## RESULTS

### Effect of rTMS on dyskinetic behavior

**Figure 1:**
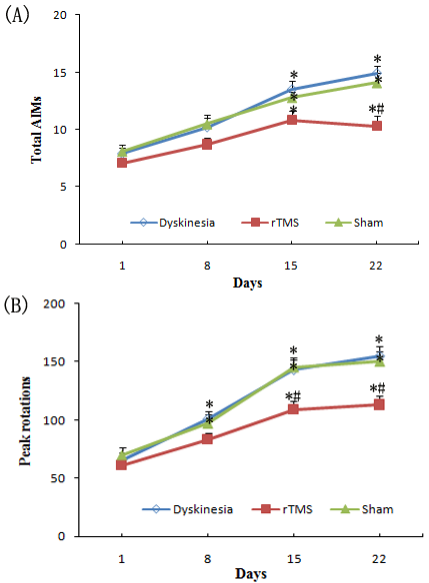
**A.** Effect of repetitive transcranial magnetic stimulation (rTMS) treatment on l-dopa-induced total abnormal involuntary movements (AIMs). **B.** Effect of repetitive transcranial magnetic stimulation (rTMS) treatment on l-dopa-induced increasing of the peak rotations. * *P* < 0.05 *versus* day 1; # *P* < 0.05 *versus* sham.

The present study showed that during the period of L-dopa treatment in PD rats for 22 consecutive days (Dyskinesia group), the total AIMs scores went up gradually. Yet, in rTMS group, rTMS treatment attenuated L-dopa-induced AIMs scores, and the effects were more obvious after three-week treatment compared with sham group (*P* < 0.05, Figure 1).

Peak rotations are another good marker for dyskinetic behavior. Similarly, peak rotations exhibited an average of 120.1±6.8% and 138.5±8.9% rise on the day of 15 and 22, respectively, than on the day of 1 after L-dopa treatment for 22 days in PD rats (Dyskinesia group). On the other hand, the PD rats treated with L-dopa plus rTMS exhibited an average of 78.7±6.6% and 83.6±9.1% more peak rotations on the day of 15 and 22, respectively, compared to day 1. Thus, L-dopa with or without rTMS increased peak rotations (*P* < 0.05), but co-treatment of rTMS significantly attenuated the increase in peak rotations (*P* < 0.05; Figure 1). Sham stimulation has no effect on dyskinetic behavior.

### Effect of rTMS on TH-positive dopaminergic neurons

**Figure 2:**
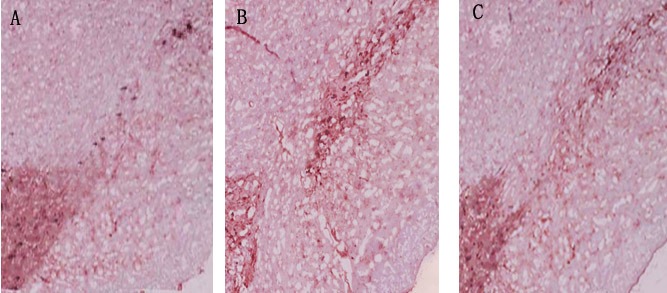
Effects of repetitive transcranial magnetic stimulation (rTMS) treatment on the number of tyrosine hydroxylase (TH)-positive dopamine neurons in the lesioned substantia nigra of the rats induced by 6-OHDA after chronic l-dopa injection on Day 22 (*n* = 3) **A.** Dyskinesia group; **B.** rTMS group; **C.** sham group.

In dyskinesia group, there was an obvious loss of TH-positive dopaminergic neuron in the 6-OHDA-lesioned ipsilateral substantia nigra. Quantitative analysis showed only 12.4±1.8% nigral TH-positive dopaminergic neurons compared with the control hemisphere. In PD rats that received L-dopa plus rTMS treatment for three weeks, there was a 25.3±3.4% more TH-positive dopaminergic neurons as compared with dyskinesia group (*P* < 0.05). Sham stimulation has no effect on nigral TH-positive neurons in dyskinetic rats (Figure 2)

### Effect of rTMS on striatal dopamine (DA) levels

**Figure 3:**
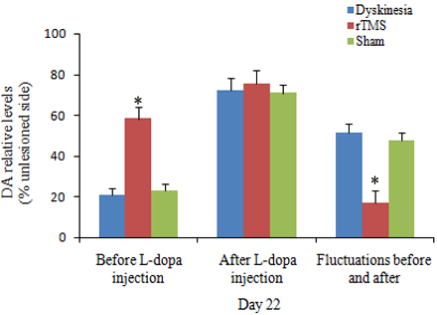
Effects of repetitive transcranial magnetic stimulation (rTMS) treatment on the relative dopamine levels in the striatum of the rats induced by 6-OHDA before (*n* = 3) and after (*n* = 3) chronic l-dopa injection on Day 22 The value of lesioned striatum is expressed as the mean percent of unlesioned striatum. *P < 0.05 *versus* sham.

Biochemical analysis of DA by HPLC in the lesioned striatal tissues was defined as a percentage of the unlesioned side. On day 22, before L-dopa administration, the relative levels of DA were 20.9±3.5%, 58.3±5.6%, and 23.1±3.2% in dyskinesia group, rTMS group and sham group, respectively. As known, single L-dopa administration increased DA levels to a peak value at 100 min. Accordingly, on day 22, at 100 min after L-dopa administration, the relative DA levels were 72.4±5.8%, 75.3±6.7%, and 70.8±4.3% in dyskinesia group, rTMS group and sham group, respectively. The fluctuations of striatal dopamine levels in rTMS group were significantly smaller than that in dyskinesia group (17.0±5.9% and 51.5 ±4.2%, respectively; *P* < 0.05) (Figure 3). Sham stimulation has no effect on DA in dyskinetic rats.

### Effects of rTMS on the expression of GDNF

**Figure 4:**
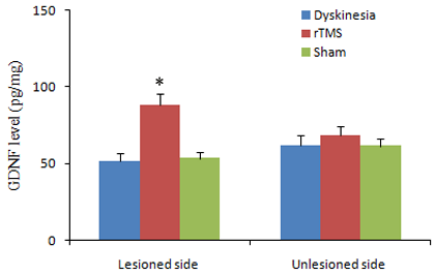
Effects of repetitive transcranial magnetic stimulation (rTMS) treatment on the expression of GDNF in the lesioned and unlesioned substantia nigra of the rats induced by 6-OHDA after chronic l-dopa injection on Day 22 (*n* = 3) *P < 0.05 *versus* sham.

To establish GDNF connected with the neurorestorative effects and the repair process of functional recovery induced by rTMS, the expressions of GDNF were detected by ELISA assay. The level of GDNF was obviously elevated in 6-OHDA-lesioned ipsilateral substantia nigra of rTMS group compared with dyskinesia group (*P* < 0.05; Figure 4). Sham stimulation has no effect on nigral GDNF in dyskinetic rats.

### Effects of rTMS on the upregulation of NR2B tyrosine phosphorylation and interactions of NR2B with Fyn in the lesioned striatum

**Figure 5:**
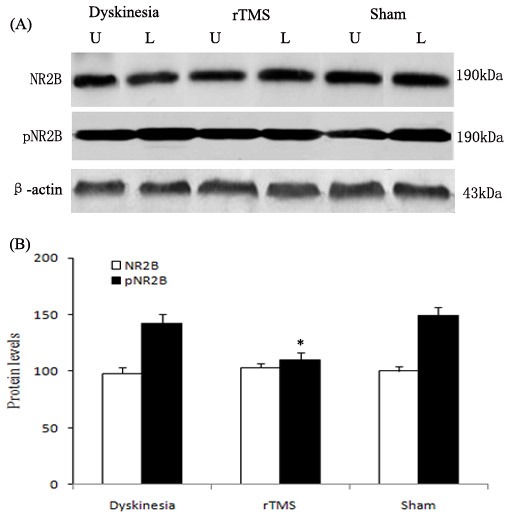
Effects of repetitive transcranial magnetic stimulation (rTMS) treatment on pNR2B-Tyr1472 and NR2B levels in dyskinetic rats Panel **A.** The band represents typical immunoblot images detected by antibodies against pNR2B-Tyr1472 and NR2B from Dyskinesia, rTMS and Sham group (*n* = 3). Panel **B.** Bands corresponding to pNR2B-Tyr1472 and NR2B on immunoblots shown as in Panel A were scanned and their optical density quantified by densitometry and the value of lesioned side expressed as percent of unlesioned side striatum. U = unlesioned side striatum, L = lesioned side striatum. *P < 0.05 *versus* Sham.

As demonstrated in Figure 5, the expression of pNR2B-Tyr1472 was upregulated obviously in dyskinesia group, and the pNR2B-Tyr1472 upregulations induced by L-dopa treatment were clearly reduced in the rTMS group (*P* < 0.05, compared with dyskinesia group). Yet, there was no significant difference in the expression of NR2B in the lesioned striatum in dyskinesia, rTMS and sham group.

**Figure 6:**
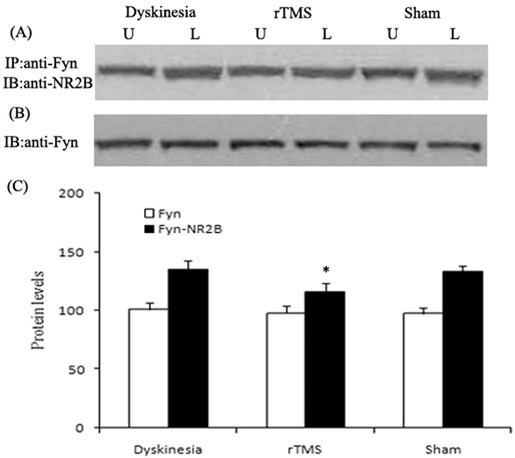
Effects of repetitive transcranial magnetic stimulation (rTMS) treatment on interactions of NR2B with Fyn in dyskinetic rats Panel **A.** Co-immunoprecipitation analysis of interactions of NR2B with Fyn. Sample proteins from Dyskinesia, rTMS and Sham group (*n* = 3) were immunoprecipitated (IP) with anti-Fyn antibodies and then blotted (IB) with anti-NR2B antibody. Panel **B.** Immunoblotting analysis of the protein levels of Fyn with anti-Fyn antibodies. Panel **C.** Bands corresponding to interactions of NR2B with Fyn and Fyn on immunoblots shown as in Panel A and B were scanned and their optical density quantified by densitometry and the value of lesioned side expressed as percent of unlesioned side striatum. U = unlesioned side striatum, L = lesioned side striatum. *P < 0.05 *versus* Sham.

Co-immunoprecipitation was used to detect the interactions of NR2B with Fyn. Similarly, as demonstrated in Figure 6, the interactions of NR2B with Fyn in the lesioned striatum following chronic L-dopa treatment, increased up to 132.6±4.9% of unlesioned side. The upregulations were clearly reduced to 117.3±6.4% of unlesioned side in the rTMS group (*P* < 0.05, compared with dyskinesia group). Yet, the Fyn expression had no differences in dyskinesia, rTMS and sham group.

## DISCUSSION

L-dopa remains the effective therapy for PD. However, It is inevitable that many PD patients develop L-dopa-induced motor complication, such as dyskinesia [1, 2]. Therefore, many studies have explored to combine L-dopa with various other treatment methods for treating PD and dyskinesia. In the present study, we combined L-dopa with rTMS treatment for dyskinesia.

The first interesting finding of the present study is that rTMS was effective in attenuating the dyskinetic behavior in chronic L-dopa-treated PD rats. Measurement of AIMs and peak rotations are the classical test performed in dyskinetic rats to assess the severity of dyskinetic behavior. In agreement with our previous report, the present study showed that L-dopa treatment for 22 days resulted in the expression of the increased AIMs and peak rotations, similarly to dyskinesia phenomena in PD patients [24]. The non-invasive rTMS has shown promising effects in improving motor disability, and might provide a therapeutic alternative with relatively few side effect [18-23]. The low-frequency rTMS treatment started with L-dopa treatment in PD rats and continued for three weeks. We demonstrated that low-frequency rTMS treatment can provide benefit on functional outcomes in behavioral test. Combination of rTMS treatment with L-dopa attenuated the increased AIMs and peak rotations to some extent, as showed in the results. These findings suggested that at least over a relative short period of time, rTMS treatment can provide adequate therapeutic benefits for dyskinesia. The mechanism of this beneficial effect was further investigated and explanated.

The second interesting finding of the present study is that rTMS could preserve dopaminergic neurons by upregulating GDNF level in chronic L-dopa-treated PD rats. As is known, previous research indicated that high-frequency (20 or 25 Hz) rTMS can activate the substantia nigra, enhance dopamine efflux in terminal areas and thus exert the beneficial effects on motor symptoms in PD rats [25, 26]. Similarly, the histological and biochemical analysis of this study also showed that low frequency rTMS-treated rats had more nigral TH-positive dopaminergic neurons and striatal dopamine level than those of the dyskinesia group and sham group. The data demonstrated that rTMS had a neuroprotective effects on survival of nigral dopaminergic neurons in chronic L-dopa-treated PD rats. We further elucidated the underlying mechanism of rTMS with such effect. As it is well known that one of the features in the PD nigrostriatal region is a severe deficiency of various neurotrophic factors (NTFs). GDNF, of the tested NTFs, was probably the most susceptible and the earliest to decrease in the survivingneuronsof substantia nigra in PD brain. Besides, thedepletionof GDNF protein level both within survivingneuronsand neuropil was greatest compared with the other NTFs [27]. Additionally, GDNF has been shown to restore the damage of dopaminergic neurons [28-30]. It was also reported that rTMS could promote the expression of NTFs [31, 32]. In the present study, we found a mild decrease of GDNF in 6-OHDA-lesioned ipsilateral substantia nigra in dyskinesia group. However, rTMS significantly increased the nigral GDNF level to higher than normal. Previous research also suggested that rTMS could change signaling pathways, gene transcription and immediate early gene expression, and initiate the biosynthesis of new molecules which persist in the tissue beyond the period of stimulation [33]. Thus, the elevation of GDNF expression by rTMS was very probably an important factor beneficial to the survival of dopaminergic neurons. More dopaminergic neurons survived and released more endogenous dopamine in the striatum. Meanwhile, much more dopaminergic neurons results in increased capacity to regulate the release of L-dopa-derived dopamine [34]. As observed in the results, rTMS could reduce the fluctuations of striatal dopamine levels before and after L-dopa administration, and thus further attenuate the abnormal reactive alteration related to dyskinesia in the striatal neurons. In this regard, the present study also provided the evidence.

As one result of the pulsatile dopaminergic receptors activating on striatal neurons, the overactivity of NMDAR function can further contribute to the expression of LID [10-12]. Recent evidences including our research reports show that the basis for the effect was that chronic dopaminergic treatment leads to the upregulation of striatal NMDAR function by Fyn-mediated NR2B tyrosine phosphorylation dependent on the interactions of NR2B with Fyn kinase [13, 14]. The present data demonstrate that the low-frequency rTMS attenuated the interactions of NR2B with Fyn and NR2B tyrosine phosphorylation, which may subsequently downregulated NMDAR overactivation, and thus offer benefit for the treatment of dyskinesia.

## CONCLUSIONS

Taken together, our data clearly indicated that rTMS may benefit for the treatment of dyskinesia in PD through rescuing the degenerative dopaminergic neurons and avoiding the fluctuations of striatal dopamine level *via* promoting the GDNF expression. Besides, the inhibition of NR2B tyrosine phosphorylation and the interactions of NR2B with Fyn may also involved in the neurorestorative effect of rTMS treatment.

## MATERIALS AND METHODS

### Subjects

Fifty adult Sprague-Dawley rats (female, 200-250 g) from Shanghai SIPPR-BK Laboratory Animal Company were selected for the study. Rats were housed under a light-controlled conditions (12/12-h light/dark cycle) with 23^o^C room temperature. Food and water and were made available. The protocol relating to animals was approved by the Local Ethics Committee and was carried out in line with the guidelines of the National Institutes of Health for the care and use of laboratory animals (NIH publication No. 80-23) and the Animals Research: Reporting *In Vivo* Experiments (ARRIVE) guidelines. The principles of the 3Rs, Replacement, Reduction and Refinement, are incorporated into guidelines and practice of animal experiments in order to safeguard animal welfare. All surgical procedures were carried out under pentobarbital anesthesia in order to reduce the amount of animals used, and their suffering.

### 6-OHDA -induced PD rat model preparation

The head of each rat was settled onto a stereotaxic apparatus (Narishige, Japan) after pentobarbital anesthesia (50 mg/kg body weight, i.p.) and received microinjections of 6-OHDA (8ug in 4 ul physiological saline). The coordinate of the right medial forebrain bundle was in line with a rat brain atlas (bregma 4.5 mm; lateral 0.9 mm and dura 7.5 mm) [35]. Three weeks after 6-OHDA microinjections, the rats that displayed apomorphine-induced rotation of over seven turns per minute away from the lesioned side were chosen for the next study [36]. 36 successfully PD rats were selected for the next research.

### Treatment protocol

Valid PD rats were divided into three groups and treated with L-dopa (25 mg/kg with benserazide 6.25 mg/kg, i.p. b.i.d.) (Dyskineisa group, *n* = 12) or L-dopa (25 mg/kg with benserazide 6.25 mg/kg, i.p. b.i.d.) plus rTMS (rTMS group, *n* = 12) or L-dopa (25 mg/kg with benserazide 6.25 mg/kg, i.p. b.i.d.) plus sham stimulation (Sham group, *n* = 12) for 22 consecutive days in order to assess the effects of concomitant rTMS plus L-dopa treatment in the prevention of dyskinesia. In sham group, the rats were given sham stimulation. In rTMS group, the rats first received treatment with repetitive magnetic stimulation, then received L-dopa treatment. The rats were placed in the epoxy holder. The magnetic stimulator from BEMS-1, China was used. The parameter of the magnetic stimulations were monophasic pulses, a figure-of-eight coil with the frequency of 0.5 Hz, daily for 3 weeks. Stimulation intensity was adjusted to 250 V/m, which was above the threshold for evoking motor responses in the hind limb muscles. A stimulation train consisted of 500 pulses. Sham stimulation was used as above described with the parkinsonian rats receiving sham stimulation, daily for 3 weeks.

### Behavioral assessment

**Figure 7:**
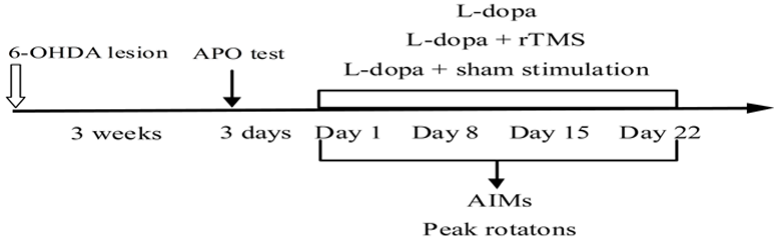
Time-course of the experiments described in the text The effect of repetitive transcranial magnetic stimulation (rTMS) treatment on the prevention of l-dopa-induced dyskinesia was studied. 6-OHDA, 6-hydroxydopamine; APO, apomorphine; AIMs, abnormal involuntary movements.

During the L-dopa treatment period, PD rats developed increasingly severe contralateral rotation and/or abnormal involuntary movements affecting cranial, trunk, and limb muscles on the side of the body contralateral to the lesion, which resembled those appearing in similarly treated parkinsonian patients. Abnormal involuntary move­ments (AIMs) were assessed according to the previous methods [37]. Briefly, on the testing day, we placed the rat separately in plastic trays five minutes before L-dopa treatment. Every rat was assessed for locomo­tor, axial, limb, and orolingual movement after L-dopa administration. At an interval of 20-minute, over a period of 3hours, AIMs were rated for sixty seconds. A sever­ity score from 0 to 4 was assigned to each category of AIMs. 0 represented AIMs absent;1 represented AIMs occasional (less than fifty percent of viewing time); 2 represented AIMs frequent (more than fifty percentof viewing time); 3 represented AIMs continuous interrupted by strong sensory stimuli; and 4 represented AIMs continuous, uninterrupted AIMs. At last, the four category of AIMs scores were summed at each time point. AIMs were measured on the day of 1, 8, 15, and 22 of treatment. Meanwhile, the peak intensity of rotation was measured as the peak number of contralateral turns in any 5-minute interval (Figure 7).

### Tyrosine hydroxylase (TH)-positive dopaminergic neurons counting by tissue immunohistochemistry

After ending the experiment, part of the rats (three rats per group) were killed after pentobarbital anesthesia (50 mg/kg body weight, i.p.). After fixed with 4% paraformaldehyde, the rat brains were removed and serial coronal frozen sections were prepared for immunohistochemistry detection. Rat brain sections were preincubated in 10% normal horse serum/0.2% Triton X-100/0.1M PBS for 1h at room temperature. After endogenous peroxidase being treated with 3% H_2_O_2_, the sections were incubated with mouse anti-rat TH antibody(1: 1000; Sigma, USA) in PBS containing 1% normal horse serum, washed with PBS, biotinylated anti-mouse secondary IgG (1:400) applied for 1h, room temperature for 30 min, washed with PBS. SABC for 20 min, washed with PBS four times, and visualized with DAB for 5min, stained with hematoxylin, then the results were observed under a light microscope (IM50, Leica).

### Dopamine measurements by high-performance liquid chromatography

Rats (three rats per group before and after L-dopa administration respectively) were sacrificed and dopamine content in the striatum was detected according to previous report [38]. Results were normalized to the sample wet weight.

### GDNF measurements by ELISA

Three rats per group were killed, then nigral and striatal tissues were removed separately. Striatal tissues were used for tissue immunoblotting. Nigral tissues were lysed using tissue lysis buffer and centrifuged at 10 000 g, 4 °C for ELISA. First, the protein concentrations in nigral samples were measured and were adjusted to the same level. Then, GDNF protein levels in samples were determined by GDNF Assay ELISA systems (Promega, USA) following the guideline of the manufacturer. The data were expressed as pg per mg of total protein. In short, 100 ul acid treated samples and standards were added to wells (96-well plate; Nunc, Naperville, IL, USA) precoated with anti-GDNF monoclonal antibody and incubated at 4°C overnight. Wells were washed and horse raddish peroxide (HRP) conjugate was added, then washed and incubated with HRP substrate tetramethylbenzidine for 15 min at room temperature. The absorbance was measured in a micro-plate reader at 450nm after adding stop solution, From the standard curves using samples with known GDNF concentrations, the GDNF concentrations were interpolated.

### Immunoblotting (IB)

Striatal tissues (three rats per group) were lysed in lysis buffer containing phe­nylmethanesulfonyl fluoride. The supernatant was collected after centrifugation at 12,000g for 10minutes at 4°C. After measurement of protein concentration using a bicinchoninic acid assay kit (Pierce, Rockford, IL, USA), samples contain­ing 20 μg protein were loaded onto 7.5% sodium dodecyl sulfate polyacrylamide gel, then transferred to PVDF after electrophoresis, blocked for 1h with 5% skim milk, and finally closed overnight with indicated antibodies, including [rabbit polyclonal anti-pNR2B-Tyr1472 antibody (1:1,000, Sigma, St. Louis, MO, U.S.A), rabbit polyclonal anti-NR2B antibody (1:1,000, Sigma, St. Louis, MO, U.S.A), mouse monoclonal anti-Fyn antibody (1:1,000, Upstate, Charlottesville, VA), or β-actin (1:1,000, abcam LTD, Cambridge, UK). (4°C, shaker)], the PVDF were washed 3 times in TBST (TBS with 0.05% v/v Tween-20) at room temperature and then incubated with horseradish peroxidase-conjugated secondary antibody diluted in TBST (1:2,000) for 1 h at room temperature followed by washing, signal detection was performed with an enhanced chemiluminiscence kit, and β-actin as an internal reference, finally analysis of the integral value of the optical density using image analysis software.

### Immunoprecipitation (IP)

After dilution four-fold with immunoprecipitation buffer, 400 ug striatal protein sample was preincubated with 25 ul protein A sepharose CL-4B (Amersham) for one hour at 4 °C to remove protein nonspecifically adhered to protein A beads. After centrifugation, the supernatant was incubated with 2 ug antibody (mouse monoclonal anti-Fyn antibody) overnight at 4 °C. 25 ul protein A sepharose CL-4B was added and the incubation continued for two hour at 4 °C. Immune complex was isolated by centrifugation and the pellet was washed three times with immunoprecipitation buffer. Bound proteins were eluted by boiling for five minutes in Laemmli sample buffer. Then, sample was centrifuged and supernatant was used for protein analysis by immunoblotting as described above.

### Data analysis

Data were demonstrated as mean ± standard error (SEM). Statistical analysis of the biochemical results were accomplished by ANOVA followed by Dunnett's *t*-test. Repeated measurements analysis of variance was used to evaluate abnormal involuntary movements. *P* < 0.05 represents statistically significant.

## References

[1] Bastide MF, Meissner WG, Picconi B, Fasano S, Fernagut PO, Feyder M, Francardo V, Alcacer C, Ding Y, Brambilla R, Fisone G, Jon Stoessl A, Bourdenx M (2015). Pathophysiology of L-dopa-induced motor and non-motor complications in Parkinson's disease. Prog Neurobiol.

[2] Thanvi B, Lo N, Robinson T (2007). Levodopa-induced dyskinesia in Parkinson's disease: clinical features, pathogenesis, prevention and treatment. Postgrad Med J.

[3] Calabresi P, Di Filippo M, Ghiglieri V, Tambasco N, Picconi B (2010). Levodopa-induced dyskinesias in patients with Parkinson's disease: filling the bench-to-bedside gap. Lancet Neurol.

[4] Sharma S, Singh S, Sharma V, Singh VP, Deshmukh R (2015). Neurobiology of l-DOPA induced dyskinesia and the novel therapeutic strategies. Biomed Pharmacother.

[5] Widnell K (2005). Pathophysiology of motor fluctuations in Parkinson's disease. Mov Disord.

[6] Kostić VS, Marinković J, Svetel M, Stefanova E, Przedborski S (2002). The effect of stage of Parkinson's disease at the onset of levodopa therapy on development of motor complications. Eur J Neurol.

[7] Obeso JA, Grandas F, Herrero MT, Horowski R (1994). The role of pulsatile *versus* continuous dopamine receptor stimulation for functional recovery in Parkinson's disease. Eur J Neurosci.

[8] Chase TN, Oh JD (2000). Striatal mechanisms and pathogenesis of parkinsonian signs and motor complications. Ann Neurol.

[9] Chase TN, Oh JD (2000). Striatal dopamine- and glutamate-mediated dysregulation in experimental parkinsonism. Trends Neurosci.

[10] Duty S (2012). Targeting glutamate receptors to tackle the pathogenesis, clinical symptoms and levodopa-induced dyskinesia associated with Parkinson's disease. CNS Drugs.

[11] Hadj Tahar A, Grégoire L, Darré A, Bélanger N, Meltzer L, Bédard PJ (2004). Effect of a selective glutamate antagonist on L-dopa-induced dyskine¬sias in drug-naïve parkinsonian monkeys. Neurobiol Dis.

[12] Papa SM, Chase TN (1996). Levodopa-induced dyskinesias improved by a glutamate antagonist in Parkinsonian monkeys. Ann Neurol.

[13] Kong M, Ba M, Liu C, Zhang Y, Zhang H, Qiu H (2015). NR2B antagonist CP-101,606 inhibits NR2B phosphorylation at tyrosine-1472 and its interactions with Fyn in levodopa-induced dyskinesia rat model. Behav Brain Res.

[14] Ba M, Kong M, Ma G (2014). Postsynaptic density protein 95-regulated NR2B tyrosine phosphorylation and interactions of Fyn with NR2B in levodopa-induced dyskinesia rat models. Drug Des Devel Ther.

[15] Oh JD, Russell DS, Vaughan CL, Chase TN (1998). Enhanced tyrosine phosphorylation of striatal NMDA receptor subunits: effect of dopaminergic denervation and L-DOPA administration. Brain Res.

[16] Dunah AW, Wang Y, Yasuda RP, Kameyama K, Huganir RL, Wolfe BB, Standaert DG (2000). Alterations in subunit expres¬sion, composition, and phosphorylation of striatal N-methyl-D-aspartate glutamate receptors in a rat 6-hydroxydopamine model of Parkinson's disease. Mol Pharmacol.

[17] Wessell RH, Ahmed SM, Menniti FS, Dunbar GL, Chase TN, Oh JD (2004). NR2B selective NMDA receptor antagonist CP-101,606 prevents levodopa-induced motor response alterations in hemi-parkinsonian rats. Neuropharmacology.

[18] Fregni F, Pascual-Leone A (2007). Technology insight: noninvasive brain stimulation in neurology-perspectives on the therapeutic potential of rTMS and tDCS. Nat Clin Pract Neurol.

[19] Lefaucheur JP (2006). Repetitive transcranial magnetic stimulation (rTMS): insights into the treatment of Parkinson's disease by cortical stimulation. Neurophysiol Clin.

[20] Yang X, Song L, Liu Z (2010). The effect of repetitive transcranial magnetic stimulation on a modelrat of Parkinson's disease. Neuroreport.

[21] Koch G (2010). rTMS effects on levodopa induced dyskinesias in Parkinson's disease patients: searching for effective cortical targets. Restor Neurol Neurosci.

[22] Sayın S, Cakmur R, Yener GG, Yaka E, Uğurel B, Uzunel F (2014). Low-frequency repetitive transcranial magnetic stimulation for dyskinesia and motor performance in Parkinson's disease. J Clin Neurosci.

[23] Filipović SR, Rothwell JC, van de Warrenburg BP, Bhatia K (2009). Repetitive transcranial magnetic stimulation for levodopa-induced dyskinesias in Parkinson's disease. Mov Disord.

[24] Henry B, Crossman AR, Brotchie JM (1998). Characterization of enhanced behavioral responses to L-DOPA following repeated administration in the 6-hydroxydopamine-lesioned rat model of Parkinson's disease. Exp Neurol.

[25] Kanno M, Matsumoto M, Togashi H, Yoshioka M, Mano Y (2004). Effects of acute repetitive transcranial magnetic stimulation ondopamine release in the rat dorsolateral striatum. J Neurol Sci.

[26] Keck ME, Welt T, Müller MB, Erhardt A, Ohl F, Toschi N, Holsboer F, Sillaber I (2002). Repetitive transcranial magnetic stimulation increases the release of dopamine in the mesolimbic and mesostriatal system. Neuropharmacology.

[27] Chauhan NB, Siegel GJ, Lee JM (2001). Depletion of glial cell line-derived neurotrophic factor in substantia nigra neurons of Parkinson's disease brain. J Chem Neuroanat.

[28] Grondin R, Gash DM (1998). Glial cell line-derived neurotrophic factor (GDNF): a drug candidate for the treatment of Parkinson's disease. J Neurol.

[29] Date I, Aoi M, Tomita S, Collins F, Ohmoto T (1998). GDNF administration induces recovery of the nigrostriataldopaminergic system both in young and aged parkinsonian mice. Neuroreport.

[30] Rosenblad C, Martinez-Serrano A, Björklund A (1998). Intrastriatal glial cell line-derived neurotrophic factor promotes sprouting of spared nigrostriatal dopaminergic afferents and inducesrecovery of function in a rat model of Parkinson's disease. Neuroscience.

[31] Dong Q, Wang Y, Gu P, Shao R, Zhao L, Liu X, Wang Z, Wang M (2015). The Neuroprotective Mechanism of Low-Frequency rTMS on Nigral Dopaminergic Neurons of Parkinson's Disease Model Mice. Parkinsons Dis.

[32] Lee JY, Kim SH, Ko AR, Lee JS, Yu JH, Seo JH, Cho BP, Cho SR (2013). Therapeutic effects of repetitive transcranial magnetic stimulation in an animal model of Parkinson's disease. Brain Res.

[33] Arias-Carrion O, Machado S, Paes F, Velasques B, Teixeira S, Cardenas-Morales L, Piedade R, Ribeiro P, Nardi AE (2011). Is rTMS an effective therapeutic strategy that can be used to treat Parkinson's disease?. CNS Neurol Disord Drug Targets.

[34] Mouradian MM, Juncos JL, Fabbrini G, Chase TN (1987). Motor fluctuations in Parkinson's disease: pathogenetic and therapeutic studies. Ann Neurol.

[35] Paxinos G, Watson C (2007). The Rat Brain in Stereotaxic Coordinates.

[36] Papa SM, Engber TM, Kask AM, Chase TN (1994). Motor fluctuations in levodopa treated parkinsonian rats: relation to lesion extent and treatment duration. Brain Res.

[37] Morgese MG, Cassano T, Cuomo V, Giuffrida A (2007). Anti-dyskinetic effects of cannabinoids in a rat model of Parkinson's disease: role of CB(1) and TRPV1 receptors. Exp Neurol.

[38] Yang J, Hu LF, Liu X, Zhou F, Ding JH, Hu G (2006). Effects of iptakalim on extracellular glutamate and dopamine levels in the striatum of unilateral 6-hydroxydopamine-lesioned rats: a microdialysis study. Life Sci.

